# Microbiology of molar–incisor hypomineralization lesions. A pilot study

**DOI:** 10.1080/20002297.2020.1766166

**Published:** 2020-05-20

**Authors:** Miguel Hernández, Paloma Planells, Eva Martínez, Alex Mira, Miguel Carda-Diéguez

**Affiliations:** a School of Dentistry, University of Barcelona, Barcelona, Spain; b School of Dentistry, Complutense University, Madrid, Spain; c Genomics and Health Department, FISABIO Institute, Valencia, Spain

**Keywords:** Demineralization, microbiology, caries, microbial ecology

## Abstract

**Objective**: An insufficient mineralization (hypomineralization) in the teeth during the maturation stage of amelogenesis cause defects in 3–44% of children. Here, we describe for the first time the microbiota associated with these defects and compared it to healthy teeth within the same subjects.

**Methods**: Supragingival dental plaque was sampled from healthy and affected teeth from 25 children with ﻿molar–incisor hypomineralization (MIH). Total DNA was extracted and the 16S rRNA gene was sequenced by Illumina sequencing in order to describe the bacterial composition.

**Results**: We detected a higher bacterial diversity in MIH samples, suggesting better bacterial adhesion or higher number of niches in those surfaces. We found the genera *Catonella, Fusobacterium, Campylobacter, Tannerella, Centipeda, Streptobacillus, Alloprevotella* and *Selenomonas* associated with hypomineralized teeth, whereas *Rothia* and *Lautropia* were associated with healthy sites.

**Conclusion**: The higher protein content of MIH-affected teeth could favour colonization by proteolytic microorganisms. The over-representation of bacteria associated with endodontic infections and periodontal pathologies suggests that, in addition to promote caries development, MIH could increase the risk of other oral diseases.

## Introduction

The bacterial communities in the oral cavity have been deeply studied since next-generation sequencing technologies became available, including organisms which are fastidious to grow [1,2]. These efforts have indicated that oral microbiota has an essential role in oral health [3]. Nowadays, the commensal and pathogenic microbiotas associated with several diseases have been established, including caries and periodontitis [4]. However, there are still some oral conditions in which a bacterial association has not been assessed. This is the case of the molar-incisor hypomineralization.

Mineralisation or maturation problems of the enamel manifest in the area of the tooth that corresponds to the developmental stage, as hard dental tissue does not have a repair mechanism [5]. The disorders that arise during the initial matrix secretory stage in the process of amelogenesis can lead to quantitative structural defects that manifest as dental hypoplasia while those affecting the maturation or mineralisation stages lead to hypomineralisation or qualitative defects [6].

**Figure 1.**
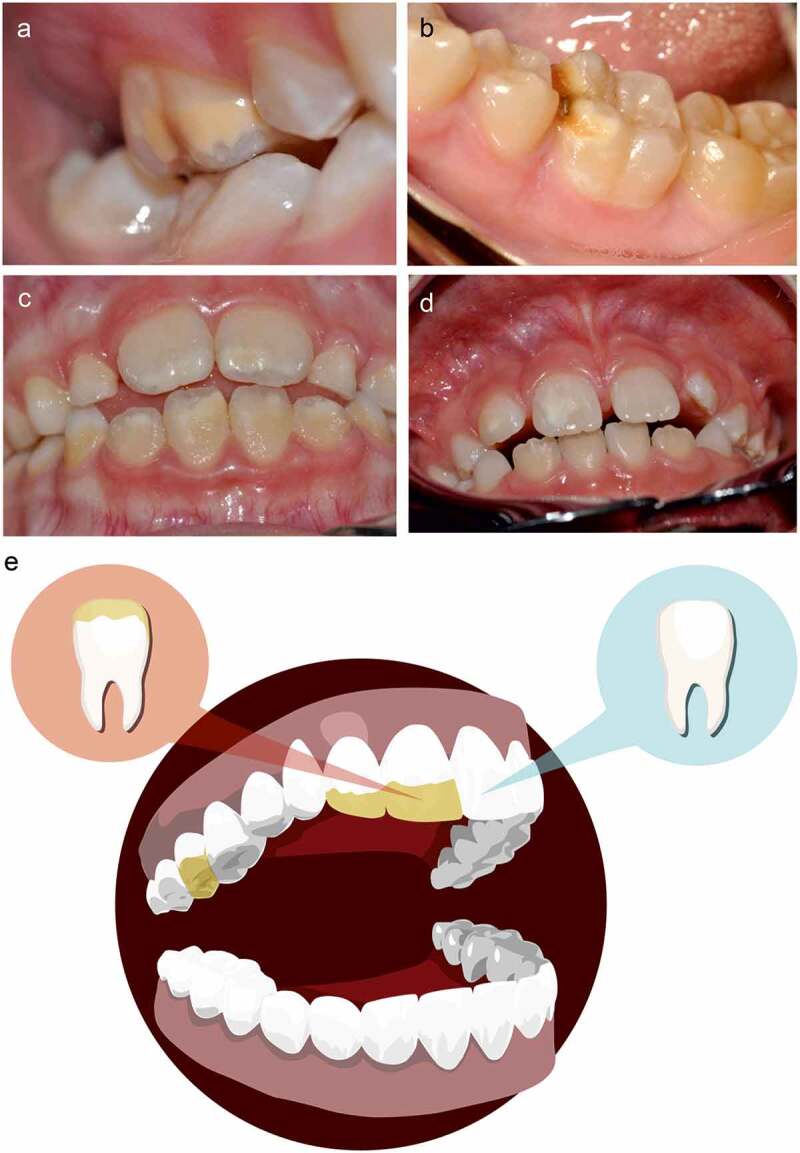
Photographic documentation of hypomineralization in representative patients. (a) Molar hypomineralization without loss of substance, brown colour, vestibular aspect of tooth #16. (b) Molar hypomineralization, with loss of substance, brown colour, mesial surface of tooth #36. (c) Incisor hypomineralization, without substance loss, brown colour, on vestibular aspect of upper and lower permanent incisors. (d) Asymmetric impact with incisor hypomineralization on the vestibular aspect of teeth #11 and #12 whereas the lesions are not observed in their homologous teeth #21 and #22, which are sampled as healthy controls. (e) Schematic representation of the sampling, including a dental plaque from the MIH-affected area and the equivalent sound aspects from the counter-lateral tooth

The term ‘molar-incisor hypomineralization’ (MIH) proposed by Weerheijm [7] was accepted during the European Academy of Paediatric Dentistry meeting held in Athens in 2003 for defining a ‘hypomineralisation of systemic origin and unknown aetiology that affects from one to four first permanent molars frequently associated with affected permanent incisors’ [8]. MIH appears as asymmetric opacities in white, cream, yellow or brown colour in the cusp or incisal third of the crown of the affected teeth, varying in extension and severity [9] (Figure 1(a–d)).

Generally, MIH develops during the first 3 years of life when the mineralization process of the crowns of the first permanent molars (FPM) and permanent incisors (PI) takes place [10,11]. Its prevalence depends mainly on the country examined (2.8% to 44% have been suggested [12]). In particular, countries with low and middle incomes and children with poor general health during the first 3 years of life, are more likely to suffer from MIH [13,14].

MIH condition can lead to serious problems such as hypersensitivity and pain, post-eruptive breakdown, chewing and eating problems, esthetics and treatment difficulties. Moreover, the poor restorative and therapeutic outcomes make MIH a challenging condition for patients, caregivers and dentists [15].

Although MIH has been found to be putatively associated with prenatal exposures to possible risk factors, a multifactorial pathogenesis with a possible genetic component is the most plausible explanation [16]. It is believed that the origin of the problem is an alteration in the resorption capacity of the organic matrix and the inhibition of the proteolytic enzymes. In consequence, proteins would be accumulated, reducing the space to deposit the minerals [10] which would result in a porous enamel [17]. In fact, MIH-affected enamel shows from 3 to 21-fold higher protein content and lower hardness and elasticity than normal teeth [18]. Consequently, the structural modification of the enamel surface creates different niches for bacterial colonization increasing the overall caries risk.

However, there are no studies in which the bacterial communities in dental plaque of MIH surfaces have been analysed. Because of the previous findings about the role that oral bacteria can have in tooth and gum destruction depending on tissue composition [4,19], and the increase in protein concentration in MIH teeth, we hypothesized that proteolytic or cariogenic bacteria could significantly be favoured in MIH-affected teeth. In order to test that, the bacterial composition and diversity of MIH teeth were determined by high-throughput DNA sequencing and compared against sound teeth in the same patients.

## Material and methods

### Sampling

The procedure to sample supragingival dental plaque from MIH patients was approved by the Bioethics committee from the University of Barcelona (ref IRB00003099). In this pilot study, 25 patients were randomly selected from a pool of 87 MIH-patients aged between 6 and 12 years old (Appendix Table A1). All of them had MIH in at least one of the first permanent molars (FPM), and all MIH teeth selected had the same degree of affection (yellow/brown opacities in the buccal aspect without PEB or atypical restorations in the rest of the tooth). The age of eruption of the affected teeth was not recorded. No selected children showed sensitivity when brushing their teeth with warm water (approximately 40ºC).

Parents/guardians of the participants were informed about the study and signed an informed consent document. Legal guardians were responsible for brushing their children’s teeth 12 hours before samples were taken. In addition, clinical data were taken for asthma, atopic dermatitis and food allergies.

After children were diagnosed with MIH, regular preventive measures were applied, both in the office and at home. From all participants, 23 individuals received fluoride twice a day in toothpaste at a concentration of 1450 ppm. Eleven children received fluoride at a concentration of 5000 ppm and 226,000 ppm in the evening brushing and every 6 months, respectively. Two participants did not receive any type of fluoride product as a demand of the legal guardians (patients 8 and 17).

Patients were appointed in the morning. They were asked to brush their teeth the night before after having dinner but not in the morning, in order to obtain a 12 h supragingival dental plaque of similar degree of maturation among donors. Each sample was collected with a different sterile excavator spoon (Figure 1(e)). The teeth were not dried before obtaining two samples from each patient: one sample was collected from an affected tooth, and the other from its contralateral counterpart (healthy control). The area sampled was similar in both control and MIH-affected teeth. The collected material was placed in a microcentrifuge tube with 500 ul of Isohelix DNA preservation solution (Cell Projects Ltd, Kent, UK) at room temperature until its processing within 30 days.

### DNA extraction and 16S rRNA gene amplification

Samples were extracted using the MagNa Pure LC DNA Isolation kit II ﻿(Roche®) and a MagNa Pure Instrument. Protocol was used as indicated by the company with some modifications following Dzidic et al. 2018 [20]. In summary, samples were lysed using 3 × 10 s cycles of ultrasounds, enzymatic digestion with an enzyme cocktail of lysozyme (100 mg/ml), lysostaphin (5 kU/ml) and mutanolysin ﻿(2.5 kU/ml). Finally, proteins were degraded using Proteinase K. DNA was resuspended in 100 ul of ultrapure DNAse-free water.

After measuring the DNA by fluorimetry, the V3-V4 hypervariable region of the 16S rRNA gene was amplified using universal primers optimized for Illumina sequencing, following Dzidic et al. 2018 [20]. Library was constructed using the Metagenomic Sequencing Library Preparation Illumina protocol (Part #15044223 Rev. A) and sequenced with the standard procedure recommended by the manufacturer, at the sequencing service in FISABIO (Valencia, Spain) using 2 × 300 bp paired-end sequencing with an Illumina MiSeq instrument. Data have been deposited in the SRA database (Bioproject PRJNA542627: Microbiology of Molar–Incisor Hypomineralization lesions, SRR9098974-SRR9099019).

### Data analysis

Dada2 was used to filter, end-trim, denoi﻿ze and merge paired reads [21]. First of all, reads were filtered for adapters and primers and then end-trimmed in 10 bp windows with quality values <35 and absence of Ns. Singletons reads were removed. The remaining reads were merged, clustered and cleaned for host and chimeric reads and, finally, assigned to a taxon using the SILVA non-redundant database [22].

Rarefaction curves, heatmaps, principal component analyses (PCoA), canonical correlation analysis (CCA) and Wilcoxon rank-sum statistical tests were performed with R, using the packages Vegan [23] and ade4 [24]. When multiple comparisons were performed, p-values were corrected by the Bonferroni correction. Statistical tests were assessed comparing each patient’s MIH sample versus its own healthy sample.

## Results and discussion

This is to our knowledge the first study of the microbial communities associated with hypomineralization. We have sequenced the 16S rRNA gene of the resident bacteria in the teeth using Illumina MiSeq in order to describe the microorganisms associated with mineralization defects of 25 patients and compare it with equivalent sound teeth from the same individuals.

Two children were excluded from the study because we were not able to obtain enough plaque material. According to their medical history, 10 individuals (7 girls and 3 boys) had atopic dermatitis; 3 children (2 boys and a girl) presented food allergies; 3 boys had asthma and two girls had celiac disease. The remaining 23 children had similar age 9 ± 1.9 year old and a similar degree of affection. Therefore, we did not consider that these two factors added significant variation to the observed results.

After quality filtering, an average of 38,112 reads per sample was obtained, and in all cases rarefaction curves reached the plateau, suggesting that the majority of samples’ diversity was sequenced.

### Microbial diversity at hypomineralized sites

**Figure 2.**
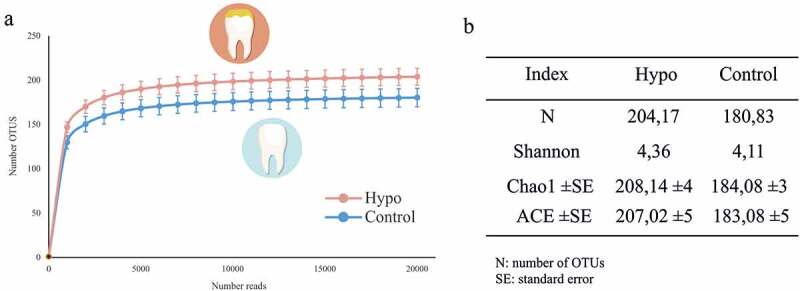
Bacterial diversity and richness analyses of microbiota associated with Healthy vs Hypomineralized teeth. (a) Rarefaction curves relating sequencing effort to the estimated number of species (OTUs at 97% sequence identity) obtained for each group. (b) Species-level diversity and richness indexes for the two groups, calculated for 20,000 reads from each sample

Interestingly, when all samples from each category (healthy or hypomineralized) were grouped, the rarefaction curve corresponding to the hypomineralized (Hypo) samples showed higher microbial diversity than the healthy, unaffected teeth (Control) (Figure 2(a)). Moreover, Shannon, Chao1 and ACE diversity and richness indexes supported this result (Figure 2(b)). Usually, when microbiotas from a healthy and a dysbiotic state are compared, a higher diversity is found in healthy samples, as it happens when comparing healthy teeth surfaces to enamel or dentin caries lesions [19]. In a few occasions, the contrary has been observed, such as in periodontal pockets vs healthy gingival sulci [25]. This has been related to higher nutrient availability or impaired immune system at the affected sites, which would facilitate microbial colonization [4]. In the case reported in the current manuscript, we hypothesize that enamel degradation would affect the porosity and therefore increase the attachable surface in the tooth and, as a consequence, it would increase the number of attached bacteria and therefore the diversity. In addition, the higher protein content in MIH lesions could also favour a larger or more diverse microbial community, particularly proteolytic organisms. The possibility that hypersensitivity on MIH teeth makes patients brush MIH-affected teeth softer or less frequently than unaffected teeth must also be considered, as this could give rise to unequal plaque accumulation.

### Microbial composition at hypomineralized sites

**Figure 3.**
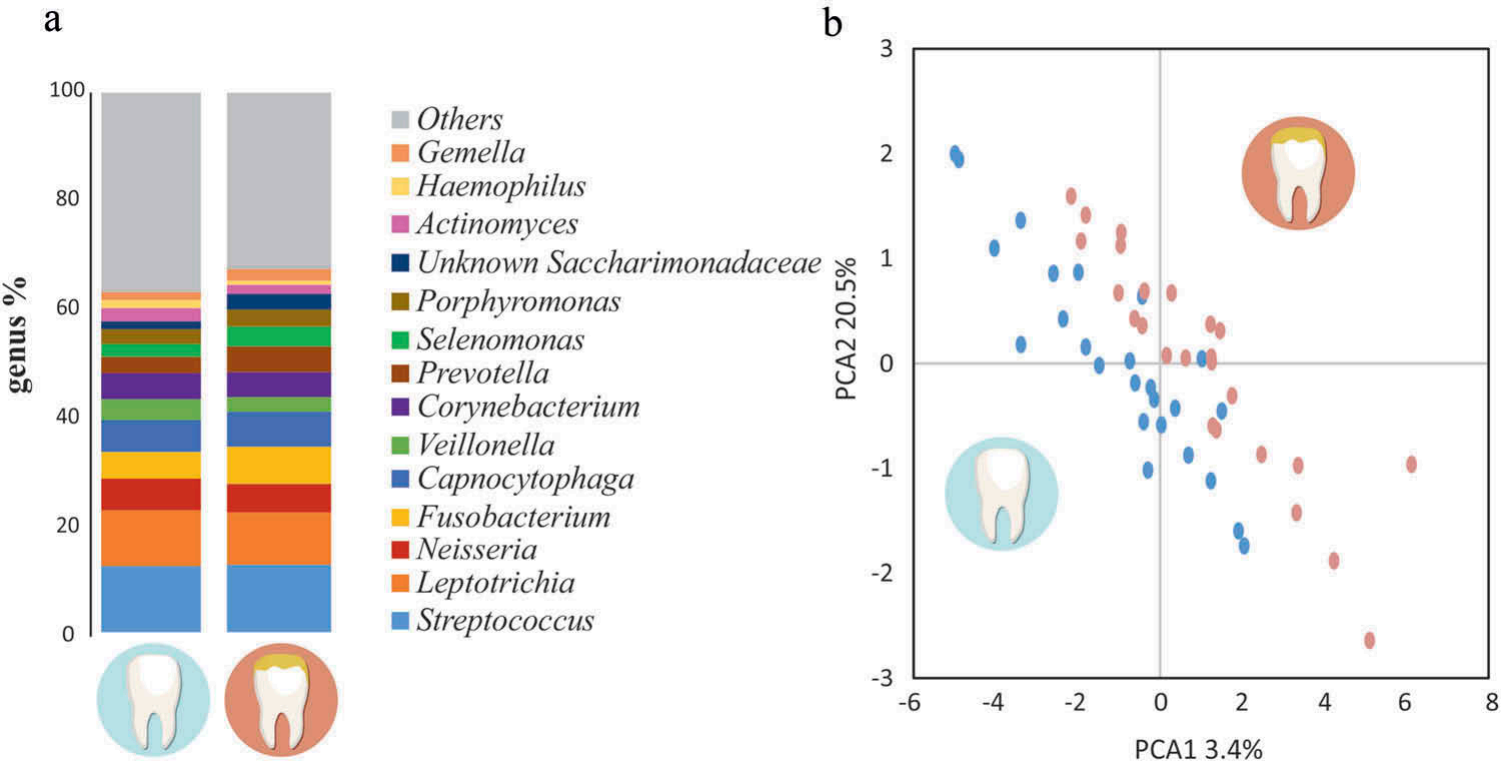
Comparison of bacterial communities associated with Healthy and Hypomineralized teeth. (a) Mean abundance of the most abundant bacterial genera (>1.5%), as determined by Illumina sequencing of the 16S rRNA gene. (b) Principal Component Analysis (PCA) performed for all samples, based on the relative abundance of each bacterial genus. The samples corresponding to healthy teeth are coloured in blue, whereas MIH-affected teeth are labelled in red

Analyses of the microbial composition at the genus level showed minor differences between the Hypo and Control regions (Figure 3(a)). In both cases, we found a highly diverse microbiota in which *Streptococcus* (12%) and *Leptotrichia* (10%) were the most abundant genus while *Neisseria, Fusobacterium, Capnocytophaga, Veillonella, Corynebacterium, Prevotella, Selenomonas, Porphyromonas* and *Saccharimonadaceae* had similar percentages (2–5%), reaching 70% of the population in both scenarios. The separation of samples in the PCoA was not significant although we could appreciate some visual differences with a trend for significance when a CCA was performed (p-value = 0.075)(Figure 3(b)).

**Figure 4.**
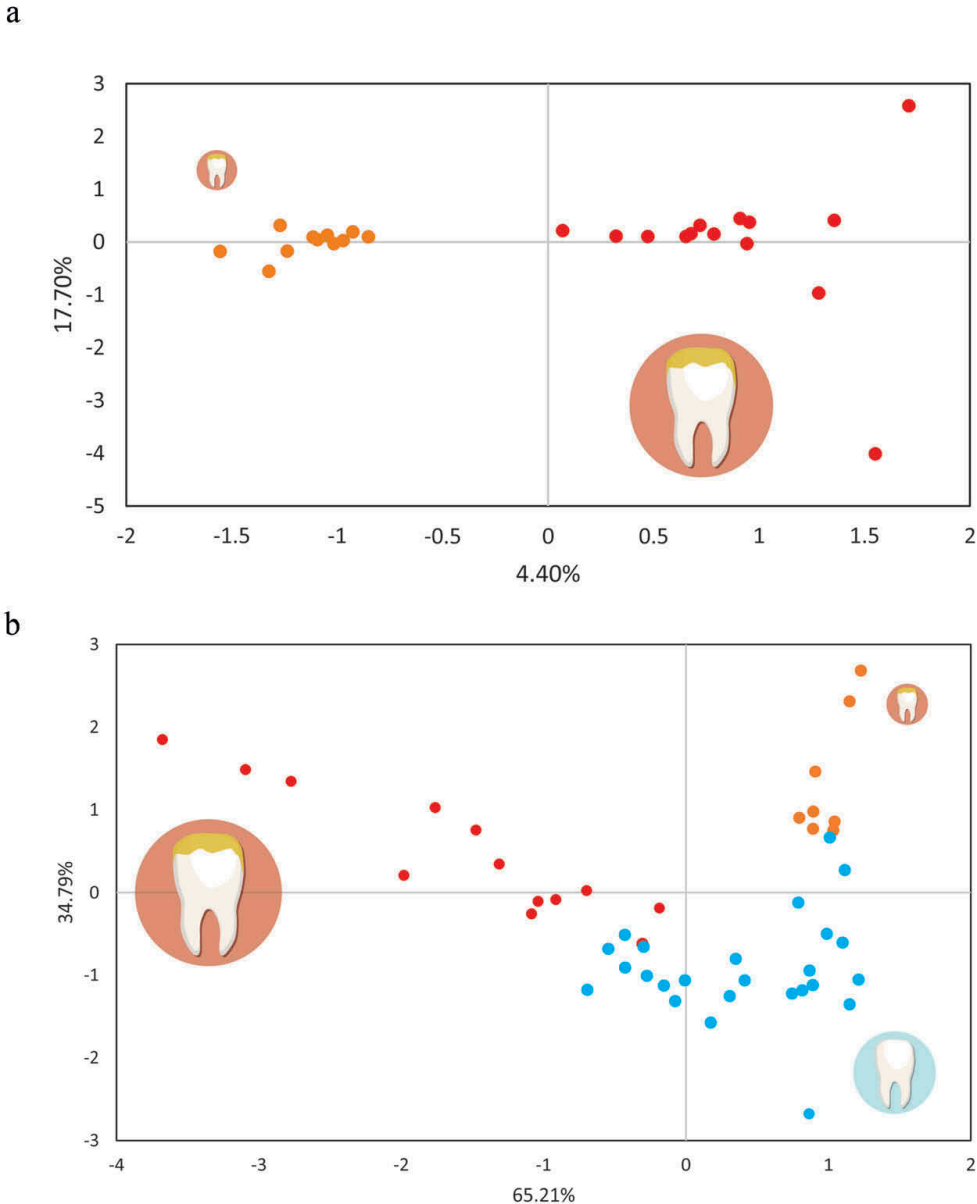
Bacterial composition (species-level OTUs) of hypomineralized teeth in individuals with different levels of hypomineralization. (a) Canonical correspondence analyses (CCA) of MIH samples. Teeth in mouths with less than 4 affected teeth were coloured in orange and with a smaller size, while those in red and close to a bigger size icon represent teeth from individuals with four or more hypomineralized teeth. (b) CCA plot comparing healthy teeth in MIH-affected mouths (blue dots), MIH-teeth in patients with less than 4 affected teeth (orange) and MIH-teeth in patients with four or more teeth affected (red)

In order to unravel the origin of these differences, we grouped Hypo samples according to several characteristics of the patients, i.e. atopic dermatitis, food allergies or asthma [10]; if they were treated with fluoride or the number of teeth affected. Only when samples were classified in two groups depending on the number of hypomineralized teeth (<3 [Low] or 4–8 hypomineralized teeth [High]), the clustering was significant (p-value = 0.04) (Figure 4(a)). This suggested that the degree of affection influenced microbial composition, but there was no correlation between bacterial composition and the other studied features of the patients’ medical history. When hypomineralized teeth from individuals with high and low MIH levels were analyzed by CCA together with their corresponding healthy controls, the secondary component of the analysis separated healthy from diseased sites, whereas negative values in the principal component were associated with affected teeth in patients with high MIH levels (Figure 4(b)). In agreement with this clustering, we detected 10 OTUs differentially represented in patients depending on the number of hypomineralized teeth (Appendix Table A2).

**Figure 5.**
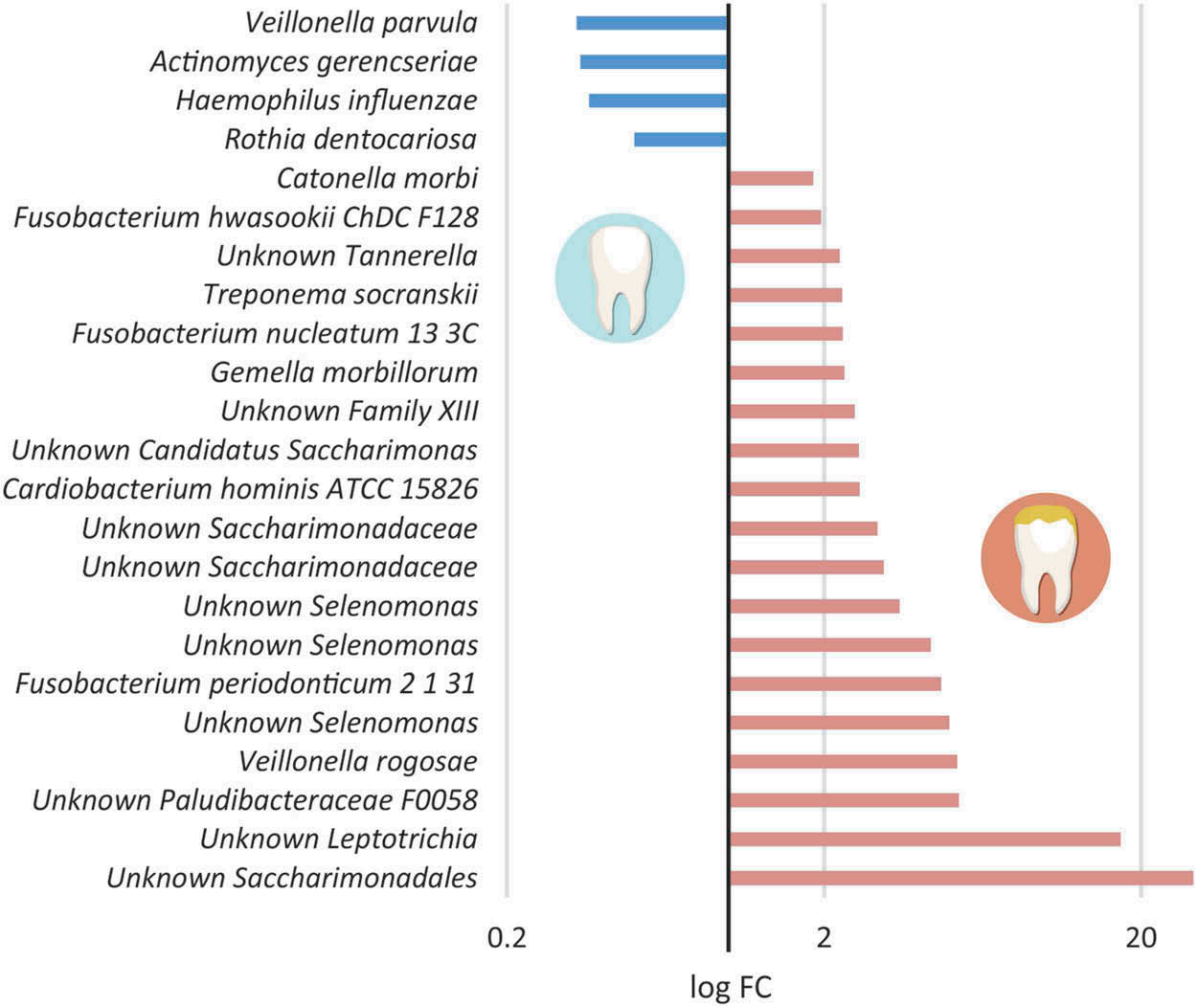
Variation in bacterial composition between hypomineralized and healthy, unaffected teeth. The fold change of those species-level OTUs which were significantly over- or under-represented in the hypomineralized samples was calculated dividing the mean abundance of each OTU in the hypomineralized teeth by its mean abundance in the healthy samples. The log_10_ values were plotted and coloured in blue and red for healthy and hypomineralized samples, respectively

Finally, we used Wilcox tests to analyze the taxons which were significantly more represented in each category (Appendix Table A3). Remarkably, *Rothia* and *Lautropia*, two genera usually associated with a healthy microbiota were the only genera associated with Control samples (p-value = 0.02). However, when we looked at the 97%-OTU (species) level, we found 6–8 members more represented in the control samples in comparison with the Hypo teeth (Figure 5, Appendix Table A4). In contrast, several taxa were significantly associated with the Hypo samples, namely: *Catonella*, unclassified members of the Clostridiales Family_XIII, *Fusobacterium, Campylobacter, Tannerella, Centipeda, Selenomonas, Streptobacillus* and *Alloprevotella*.

From those, *Streptobacillus* and the members of the Clostridiales Family XIII are the only taxa with species not previously associated with oral diseases. In contrast, species from the rest of the genera such as *Catonella morbi, Tannerella forsythia, Selenomonas spp*. or *Fusobacterium* sp. OT203 have been associated with ‘refractory’ periodontal patients, i.e. those who do not respond to conventional therapies and where loss of periodontal attachment is not reversible [26]. On the other side, Colombo and collaborators detected a significant prevalence of *Haemophilus, Capnocytophaga, Lautropia, Veillonella* and *Rothia* in patients with therapeutic success, all of which were more abundant in our Control samples.

Moreover, we found other examples of studies that associated the Hypo-related species with periodontitis. For example, *Centipeda periodontii* has been associated with periodontitis in Taiwanese patients [27]. Siqueira and Roças found *Catonella morbi* in 33% of root canals in chronic apical periodontitis and in 26% of primary endodontic infections by amplifying the 16S rRNA gene [28]. Finally, in two studies using next-generation sequencing technologies in which the microbiota of healthy and periodontitis patients was compared, a significant abundance of *Catonella* was detected in those affected [29,30].

In addition, *Campylobacter, Centipeda, Alloprevotella, Fusobacterium, Selenomonas* and *Catonella* were part of the 17 taxa significantly enriched in the oral samples of patients with oral cancer [31]. In this same study, Zhao et al. found *Lautropia, Veillonella, Rothia* and *Actinomyces* associated with controls. It has to be kept in mind that most bacterial species found to be associated with hypomineralized lesions were highly proteolytic, suggesting that the higher protein content at those sites may favour the growth of these bacteria, which happen to be associated with various oral and systemic diseases.

In conclusion, although the differences between healthy and hypomineralized teeth are small, there are some taxa which appear to be significantly associated with each situation. Although we do not have samples from healthy individuals in our study, the differences detected depending on disease severity suggest that MIH may select for disease-associated bacteria and that contrary to our expectations, those were not saccharolytic and therefore related to caries risk. Furthermore, most taxa found in this study to be associated with hypomineralized teeth have previously been related to other oral diseases. On the contrary, bacteria found to be associated with healthy teeth unaffected by MIH have been found in other studies more abundantly when these diseases are absent. This suggests that MIH could be related not only to caries progression due to reduced mineral content but also to a higher risk of periodontal diseases because of higher levels of bacteria associated with this condition. Thus, we suggest that future research, especially longitudinal studies, should investigate if the MIH-associated pathogenic communities can increase the risk of other oral and systemic diseases which are influenced by proteolytic microorganisms. In addition, further studies should clarify if the pathogenic community associated with MIH teeth is only a consequence of the disease or whether it can contribute to its progression. For instance, the proteolytic bacteria detected in the current manuscript could degrade the high protein content of MIH-affected teeth, facilitating the microbial invasion of dentinal tubules, which has been demonstrated using electron microscopy [32], and contribute to cavitations and hypersensitivity.

## Appendix

**Table A1.** Patients’ dataset

Patient	Sex	Condition	Age	Sampled teeth	HIM teeth	Fluor treatment (ppm/type)
1	F	– –	10	16/26	16,21,36,31,46	1450/5000
2	M	AD	8	16/46	16,26,31,11,21,36	1450/22,600
3	F	– –	12	16/36	16,26,11,21,41,46	1450/22,600
4	M	– –	8	16/26	16,11,21,36,31,41,46	1450/22,600
5	F	AD	6	16,26/36	16,26,46,31,41	1450
6	F	– –	12	46/26	16,46	1450
7	F	AD	8	16/26	16,36,46	1450/22,600
8	M	FA, ASMA	8	16/26	16,36,46,21	-
9	F	AD,FA	8	16/36, 46	16,11,21,26,36oc,32,31,46oc	1450/5000
10	M	ASMA, FA	11	16/36	16,11,26	1450/5000
11	F	AD, FA	8	16, 26/55,65	16,26,36,46	1450/5000
12	F	– –	6	16/26, 65	16,26,36,46, 11,21,31,41	1450
13	F	AD	11	16/26	16,46	1450
14	F	– –	9	26/36	26	1450/5000
15	M	– –	7	26/16	26,36,46	1450/5000
16	M	ASMA	11	36, 26/16, 15	26,36	1450/5000
17	F	AD, FA	8	26/36	11,21,26,31,41,42,46	-
18	M	– –	8	36/16	21,26,36,46	22,600
19	M	AD	12	26/16	26,36,46	1450
20	F	AD	11	26/16	26,36,46	1450/5000
21	F	– –	10	26/36	16,26,46	1450/5000
22	M	– –	10	16/26	16,36,46	1450/5000
23	M	AD	7	16/26	16,36,46,41,32	2500

F, female; M, male; AD, atopic dermatitis; FA, food allergies.

M, male

AD, atopic dermatitis

FA, food alergies

**Table A2.** OTUs differentially represented in patients depending on the number of hypomineralized teeth

OTU	p-value	Low	High	ID
OTU-16	0.0059	2.049	0.047	*Unknown Actinomycetaceae F0332*
OTU-10	0.0137	1.2	0.621	*Lautropia mirabilis*
OTU-206	0.03	0.268	0.02	*Streptococcus gordonii*
OTU-150	0.0346	0.213	0.023	*Unknown Leptotrichia*
OTU-26	0.042	0.437	0.901	*Unknown Tannerella*
OTU-38	0.0059	0.133	1.099	*Gemella morbillorum*
OTU-61	0.036	0.147	0.651	*Capnocytophaga ochracea*
OTU-335	0.03	0.007	0.066	*Unknown Leptotrichia*
OTU-365	0.036	0.009	0.134	*Unknown Saccharimonadales*
OTU-502	0.036	0.002	0.05	*Unknown Selenomonas*

Low; 11 samples with less than 4 teeth with MIH.

High, 14 samples 4 or more teeth with MIH.

**Table A3.** **T**axons significantly more represented in control and MIH samples

Taxon	p value	Mean control	Mean Hypo
*Catonella*	0.0075	0.1019	0.1874
*Clostridiales Family_XIII*	0.0144	0.0223	0.0658
*Fusobacterium*	0.0163	5.6856	7.7603
*Centipeda*	0.0238	0.0263	0.2071
*Rothia*	0.0254	2.1623	1.2803
*Tannerella*	0.0277	0.7320	1.2140
*Campylobacter*	0.0484	1.3786	1.8928

**Table A4.** Operational taxonomic units (97% identity) significantly more represented in Control and MIH samples

taxon	p.value	mean Control	mean Hypo	ID
OTU-124	0.0440	0.1012	0.2621	*Cardiobacterium hominis ATCC 15,826*
OTU-144	0.0346	0.0153	0.0717	*Fusobacterium periodonticum 2 1 31*
OTU-155	0.0052	0.1004	0.1852	*Catonella morbi*
OTU-157	0.0454	0.0483	0.2566	*Unknown Paludibacteraceae F0058*
OTU-17	0.0239	0.9613	0.4854	*Rothia dentocariosa*
OTU-189	0.0415	0.0440	0.1521	*Unknown Selenomonas*
OTU-232	0.0209	0.0451	0.1030	*Fusobacterium nucleatum 13 3 C*
OTU-247	0.0288	0.0396	0.0903	*Treponema socranskii*
OTU-26	0.0117	0.3208	0.7185	*Unknown Tannerella*
OTU-29	0.0145	0.9729	0.3313	*Actinomyces gerencseriae*
OTU-292	0.0300	0.0304	0.0897	*Unknown Saccharimonadaceae*
OTU-35	0.0423	0.2800	0.8641	*Unknown Saccharimonadaceae*
OTU-365	0.0225	0.0024	0.0685	*Unknown Saccharimonadales*
OTU-377	0.0211	0.0218	0.0544	*Clostridiales Family XIII*
OTU-379	0.0360	0.0188	0.0482	*Unknown Candidatus Saccharimonas*
OTU-38	0.0115	0.2616	0.6063	*Gemella morbillorum*
OTU-437	0.0129	0.0035	0.0595	*Unknown Leptotrichia*
OTU-50	0.0411	0.2790	0.5448	*Fusobacterium hwasookii ChDC F128*
OTU-502	0.0249	0.0064	0.0318	*Unknown Selenomonas*
OTU-515	0.0249	0.0071	0.0306	*Unknown Selenomonas*
OTU-598	0.0225	0.0361	0.0000	*Unknown Leptotrichia*
OTU-639	0.0143	0.0047	0.0248	*Veillonella rogosae*
OTU-75	0.0465	0.4545	0.1508	*Veillonella parvula*
OTU-8	0.0354	1.6551	0.6008	*Haemophilus influenzae*
